# Incidence and Risk Factors of Retinopathy of Prematurity in Mashhad, Northeast Iran

**DOI:** 10.5812/ircmj.4513

**Published:** 2013-03-05

**Authors:** Majid Abrishami, Gholam-Ali Maemori, Hassan Boskabadi, Zakiye Yaeghobi, Shahin Mafi-Nejad, Mojtaba Abrishami

**Affiliations:** 1 Eye Research Center, School of Medicine; Mashhad University of Medical Sciences, Mashhad, IR Iran; 2 Neonatal Research Center, Ghaem Hospital, School of Medicine, Mashhad University of Medical Sciences, Mashhad, IR Iran; 3 Neonatal Research Center, Ghaem Hospital, School of Medicine, Mashhad University of Medical Sciences, Mashhad, IR Iran; 4 Eye Research Center, Mashhad University of Medical Sciences, Mashhad, IR Iran; 5 Department of Pediatrics, Ghaem Hospital, School of Medicine, Mashhad University of Medical Sciences, Mashhad, IR Iran; 6 Eye Research Center, School of Medicine; Mashhad University of Medical Sciences, Mashhad, IR Iran

**Keywords:** Retinopathy of Prematurity, Risk Factors, Infants, Premature, Infants, Very Low Birth Weight, Infants, Newborns

## Abstract

**Background:**

Retinopathy of prematurity (ROP) is a vascular retinal disease that can cause low vision or blindness and affects premature newborns of very low birth weight.

**Objectives:**

The purpose of this study was to determine the incidence and risk factors for ROP among very premature infants in Mashhad located northeast of Iran.

**Material and Methods:**

In this cross-sectional study performed between 2006 and 2010, predisposing factors and severity of ROP were evaluated in very premature infants (<32 gestational weeks). Consecutive infants were enrolled at birth and screened for ROP at 4 to 6 weeks of age by indirect ophthalmoscopy. Severe ROP was defined as stage 4 or 5. Chi-square, Student’s t-, and Fisher’s test were used for statistical analysis.

**Results:**

The incidence of ROP was 26.2%. Significant differences between the ROP and control groups were observed, these include; gestational age, sex, birth weight, Apgar score, durationof parenteral nutrition, oxygen therapy, phototherapy, maximum PaO2 and minimum SpO2 (P < 0.05). Severe ROP was seen in 31.4% (11/32) of ROP cases (5.4% of newborns).

**Conclusion:**

The incidence of ROP is relatively high in this region. Risk factors for ROP among very premature infants include hypoxia, severe hyperoxia, relatively low blood SPO2, gestational age, birth weight, and Apgar score.

## 1. Background

Retinopathy of prematurity (ROP) is a disease which affects premature newborns with very low birth weight and it can result in low vision and in severe cases blindness. Early diagnosis could improve the success rate of treatment and reduce complications of the disease. Many studies worldwide have focused on the incidence of ROP, owing to the increasing survival rates among premature infants. The incidence of ROP remains low in poor countries, due to the low survival rate of premature infants (1). The improvement of neonatal care in Iran has increased the survival rate of premature infants and consequently, the incidence of ROP. Therefore, it is necessary to clarify the ROP incidence in different parts of the country. As far as we know, there is no information available about the characteristics of ROP in Mashhad, northeast Iran.

## 2. Objectives

The goal of this study was to evaluate the incidence and risk factors of ROP in very premature infants born from 2006 to 2010 in Mashhad.

## 3. Materials and Methods

This cross-sectional study was performed from May 2006 to April 2010. Newborns admitted to the neonatal intensive care unit (NICU) at Ghaem Hospital, a tertiary referral hospital in Mashhad, at a gestational age of < 32 weeks were enrolled in this study. Gestational age was determined on the basis of the Balard table, fetal sonography within the first trimester or last menstrual period (LMP). Screening for ROP commenced between 4 and 6 weeks after birth. At 30 minutes before an infant was examined, 1 drop of cyclopentolate hydrochloride (2 mg/mL) and phenylephrine (2.5 mg/mL) was instilled into each eye to obtain mydriasis. A single investigator (MA) examined the fundi using an indirect ophthalmoscope and a 20D lens in Khatam al Anbia Hospital, a tertiary referral eye hospital. Examinations were continued until the retina was fully vascularized or the infant reached a postconception age of 40 weeks, as determined by an accurate gestational age. If ROP existed, then treatment was performed according to the severity and diagnostic criteria. If primary retinal examination of the newborn was normal, then the infant was selected for the control group. This study was confirmed by the ethics committee of Mashhad University of Medical Sciences. All parents provided written informed consent. The following parameters were examined and recorded: gestational age, episodes of hypoxia based on pulse oxymetery and serial Arterial Blood Gas (ABG), rates of blood transfusion and exchange, duration and method of oxygen therapy, gender, multigravity, bilirubin value, phototherapy, underlying disease, duration of total parenteral nutrition (TPN), cerebral hemorrhage, chronic lung disease, sepsis, hypothermia, pneumothorax, jaundice, pulmonary hemorrhage, Apgar score, PO2, PCO2 and SPO2 levels. Data from the newborns, including primary examination, changes in condition during hospitalization and laboratory values were recorded. Maternal data including history of preeclampsia and hypertension, infertility, cerclage and premature rupture of membrane (PROM) were assessed and recorded by a questionnaire. Data were recorded with the SPSS software package. Chi-square test was used to analyze qualitative variables. Student’s t-test and Fisher’s test were used for quantitative variables. The coefficient of confidence of this study was 0.95. A difference with P < 0.05 was considered statistically significant.

## 4. Results

**Figure 1.**
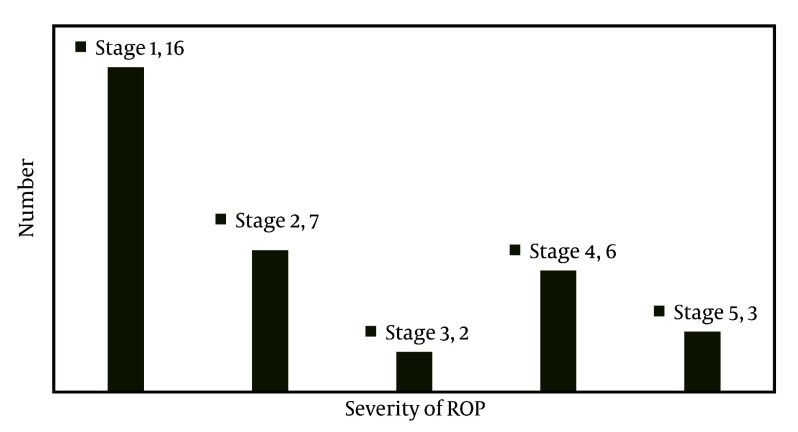
Severity of retinopathy of prematurity

Approximately 960 premature (< 37 weeks) newborns were admitted to the NICU over the 4 years of the study, including 314 neonates at gestational age of < 32 weeks. Although primary examination and questionnaire completion was performed for 304/314 of newborns, 110 newborns died. A total of 151 newborns were referred for ophthalmic examination, but 29 newborns were excluded from the study due to improper follow-up. Therefore, 122 newborns (65 female, 57 male) were evaluated by a retinal surgeon and were included in the study. The mean gestational age, birth weight, and 5 minute Apgar score for these infants were 30.54 weeks, 1249 g, and 7.30 respectively. Of the 122 newborns, 51 newborns were delivered vaginally (Table 1). Newborns were categorized into two groups: case (ROP occurred) and control (normal). In 32 newborns (26.2%), ROP was diagnosed at the initial examination. The mean gestational ages in the case and control groups were 29.19 ± 2.12 weeks and 31.16 ± 1.95 weeks, respectively (P = 0.003). The mean Apgar scores were 6.6 and 7.6, respectively (P = 0.001). Compared to the control group, the case group showed a higher mean bilirubin level and longer mean durations of phototherapy and TPN (Table 2). We found that ROP was more common among boys than girls and was frequently associated with internal cerebral hemorrhage. In the case and control groups, the maximum oxygen saturation values by pulse oxymetery were 96% and 91.7%, respectively (P = 0.534), and the minimum oxygen saturation values were 63.8% and 84.26%, respectively (P = 0.000). There was no significant difference for pH, PCO2, delivery type or intrapartum complications between the groups. Oxygen was required by 31 newborns (97%) in the case group and 46 newborns (65%) in the control group (P = 0.03). Among newborns with ROP, 13 newborns (42%) received oxygen by oxyhood and 18 newborns (57%) received oxygen by CPAP or ventilator. In the control group, oxygen consumption through oxyhood and mechanical ventilator was reported in 13 newborns (14%) and 14 newborns (16%), respectively. The ROP severity is summarized in Figure 1. Operation was performed for 6 patients in stages 3 and 4. Anatomical success was achieved in 2 patients while the other 4 newborns did not show improvement. Three cases were inoperable. Internal cerebral hemorrhage occurred in 4 newborns with ROP. There was no significant difference between cases and controls for the incidence of pneumonia (P = 0.1), patent ductus arteriosus (P = 0.26), respiratory distress syndrome (P = 0.053) or necrotic enterocolitis (P = 0.90). Moreover, ROP development was not significantly associated with delivery type, preeclampsia, abortion, cerclage, meconium aspiration, PROM, multigravity, sterility, apnea, ventilation, PDA, pneumonia or convulsion (P > 0.05).

**Table 1.** Demographic and Prenatal Characteristics of Infants Chi-square test

Variable	Group		P value
	Case	Control	
**Sex, No.**			0.007
Male	23	34	
Female	11	56	
**Mode of delivery, No.**			0.665
ND	16	38	
CS	18	52	
**Multiple pregnancy, No. (%)**	14(34)	37(100)	0.671
**Pregnancy complication, No. (%)**	9(34)	49(100)	0.108
**Intraventrcular Hemorrhage, No. (%)**	12(34)	6(81)	0.010

**Table 2.** Neonatal Characteristics of Case and Control Groups

Variables, Mean ± SD	Group		P value
	Control	Case	
**Gestational age, week**	1.95±31.16	2.12±29.12	0.001
**Weight, gram**	227±1295.6	226±1144.8	0.003
**Apgar score**	0.9±7.6	1.5±6.6	0.001
**Duration of TPN, day**	4±5	5.6±9	0.040
**Bilirubin, mg/dl**	3.7±9.2	5.0±10.4	0.281
**Duration of phototherapy, day**	3.4±8.5	5.5±12.3	0.001
**Mean of Spo2**	18±91.7	4±96	0.534
**Maximum of PO2**	1.7±96.5	42±140.8	0.020
**Minimum of SPO2**	8.6±84.26	12.1±63.8	0.000
**Mean of Pco2**	7.6±37.7	8.2±38	0.161
**Mean of PH**	0.04±7.33	0.08±7.31	0.591
**Duration of Oxygen therapy**	4.8±5.8	6.3±9.2	0.015

## 5. Discussion

The American Academy of Ophthalmology, American Academy of Pediatrics, and American Association of Pediatric Ophthalmology and Strabismus have recommend ROP screening for infants with a birth weight of < 1500 g or gestational age of ≤ 30 weeks and with a birth weight of 1500–2000 grams or gestational age of > 30 weeks with an unstable clinical course (2). The United Kingdom National Guidelines recommended ROP screening for babies with birth weight of < 1501 g or gestational age of < 32 weeks (3). In this case series, we evaluated the ROP incidence and risk factors in a tertiary hospital in Mashhad, enrolling infants at gestational age of < 32 weeks. We observed an ROP incidence of 26.2% among very premature and very low birth weight infants. Previous studies have reported ROP incidence in Newslands (21.5%) (4) , India (27%) (5) , Norway (10%) (6) , Oman (34%) (7) , Finland (27.3%) (8) , Australia (16%) (9) and Singapore (29.3%) (10) as well as in other parts of Iran, including Foladinegad (5.6%) (11), Nakhshab (11.7%)(12) and Mansouri (29.9%) (13). Differences in the incidence rates in Iran may be due to different conditions of the NICUs where the studies were performed. Lower incidence may be related to the admission of higher gestational age infants who had fewer problems. Our results are similar to those of an investigation carried out in Tehran (13); this may be explained by the similar facilities and relatively equal levels of prenatal care at the centers. In a recent study performed in Babol, northern of Iran, the incidence of ROP was 47.3% for infants with gestational age of ≤ 32 weeks and 33.3% for infants with birth weight of < 1500 g, whereas ROP developed in 2 infants (6%) with birth weight of > 2000 g (14). Karkhane et al. found that 34.5% of premature infants had different stages of ROP. Severe ROP was seen in 22.6% of infants, of whom 16.5% were treatable and 6.1% were advanced/ untreatable (15). Although the incidence has improved in recent years, it is less than that reported in these studies. In the current study, the mean gestational age and birth weight were 30.5 weeks and 1249 ± 276 g, respectively. Previous studies have reported mean gestational ages of 29.7 weeks (10) and 29.1 weeks (16). Our findings indicate that ROP was more common among patients with lower gestational age, birth weight, and Apgar score. The mean gestational age and birth weight in the ROP group were 2 weeks and 150 grams less than those in the control group, respectively (P = 0.003). The strong relationship between ROP and lower gestational age is logical, on the basis of the pathophysiology of ROP. A prerequisite for ROP is the incomplete growth of retinal vessels in premature newborns. Preterm newborns are more exposed to risk factors such as hypoxia and hyperoxia than term newborns. We suggest that ROP evaluation should be performed in all infants with gestational age of < 32 weeks because most cases of ROP occurred among neonates born at 28–32 weeks. The Apgar score in ROP newborns was lower than that of newborns without ROP (P = 0.001). The association between asphyxia and ROP incidence was expected, considering that hypoxia and oxidant production are more common during asphyxia (17). The phototherapy duration in the ROP group was significantly longer than that in the control group as observed in previous studies (18, 19). Oxygen free radicals play an important role in ROP development. Serum bilirubin within the first days of life has antioxidant effects. By reducing the levels of this antioxidant, phototherapy is likely to intensify ROP. Therefore, the indications for phototherapy use (and especially prophylactic phototherapy) in preterm infants should be revised and carefully followed. Phototherapy light might also be associated with ROP development, although this hypothesis has not yet been proven (18, 19). We found a significant relationship between maximum blood PO2 (P = 0.020) and minimum blood SPO2 (P = 0.000) and ROP incidence. Although several studies have reported correlations between hypoxia and hyperoxia with ROP, the maximum and minimum rates were not sufficiently identified (20, 21). In light of the results of this study, hypoxia, extreme hyperoxia, and low blood SPO2 can be defined as predisposing factors for developing ROP. We also observed a significant relationship between the duration of O2 therapy and ROP incidence. The results indicated that longer hypoxia occurred among the newborns developing ROP. Similar studies in Malaysia, China, and Iran have reported the same results (5, 8, 13). Hossein et al. showed that the gestational age and duration of oxygen therapy were useful values for predicting ROP (22). A study by Goble et al showed significant relationships between ROP incidence and low birth weight, early gestational age, patent ductus arteriosus, IVH, CLD, neonatal convulsion, prolonged oxygen therapy, mechanical ventilation, CPAP and pre-eclampsia (16). In animal studies, abnormal retinal response to variable oxygen therapy was shown among premature newborns (13). Human studies have indicated that prevention of hypoxia and hyperoxia decreases ROP incidence (11, 13). Therefore, the appropriate use of oxygen (particularly during resuscitation and ventilation), with consideration of the probable side-effects, may decrease the incidence of adverse ophthalmic events in premature newborns. Advanced ROP (stages 4 and 5) was found in 5.4% of newborns. Similarly, Hossein et al. found that the incidence of intensive ROP was 4.6% (22). Other studies have reported an incidence of advanced ROP of 0.6%. Mansouri et al observed that advanced ROP occurred in 1.4% of cases (13). The reason for the difference between the reported incidence rates may be due to the wide spectrum of gestational ages used in the studies. We found that ROP incidence has increased by approximately 6.5 times compared to the previous 12 years at this center (23) (4% during 1998–2000 vs. 26.2% during 2006–2010). These results emphasize the improvement of prenatal care and survival rate of babies born at this center in the last 12 years. In conclusion, the incidence of ROP is relatively high in the region of Mashad in the northeast of Iran. This incidence is different from that in other parts of Iran and has significantly increased during the last decade. Hypoxia, extreme hyperoxia, low blood SPO2, relatively low gestational age, birth weight and Apgar score are risk factors for ROP among very premature infants.
